# An Integrated Approach to Chronic Low Back Pain: Evaluating the Impact of Consecutive Loop TheraBand Training Combined With Proprioceptive Neuromuscular Facilitation and Conventional Physiotherapy

**DOI:** 10.7759/cureus.58632

**Published:** 2024-04-20

**Authors:** Priya Tikhile, Deepali S Patil, Pratik R Jaiswal

**Affiliations:** 1 Musculoskeletal Physiotherapy, Ravi Nair Physiotherapy College, Datta Meghe Institute of Higher Education and Research, Wardha, IND; 2 Sports Physiotherapy, Ravi Nair Physiotherapy College, Datta Meghe Institute of Higher Education and Research, Wardha, IND

**Keywords:** multimodal pain management, theraband training, proprioceptive neuromuscular facilitation, physical therapy, chronic low back pain

## Abstract

Chronic low back pain (CLBP) is a prevalent musculoskeletal condition characterized by persistent discomfort in the lumbosacral region lasting beyond 12 weeks. Individuals with CLBP often experience limitations in range of motion and compromised performance of affected body parts. Core muscle weakness/delayed activation and impaired lumbar proprioception are established contributors to CLBP. And influence balance dysfunction in CLBP patients. Exercise therapy is a cornerstone in the management of CLBP, aimed at enhancing muscular endurance, strength, and flexibility of the back muscles and soft tissues. However, the efficacy of exercise interventions depends on various factors including the type, intensity, frequency, and duration of exercises. This case report presents the rehabilitation of a corporate employee with a non-specific CLBP. The rehabilitation goals focused on improving balance, reducing disability, and alleviating pain. An integrated approach combining proprioceptive neuromuscular facilitation (PNF) with Consecutive Loop TheraBand (CLX) (The Hygenic Corporation, Akron, USA) along with traditional physical therapy techniques was implemented. PNF, a well-established technique, was chosen for its effectiveness in reducing disability and LBP while enhancing balance. The integration of PNF alongside conventional physiotherapy resulted in notable improvements, including increased lumbar flexion range following the rehabilitation period. This case underscores the importance of early initiation of comprehensive rehabilitation in CLBP patients to preserve strength, alleviate pain, reduce functional disability, and enhance balance. By addressing both the muscular and proprioceptive aspects of CLBP, this integrated approach aims to optimize outcomes in CLBP management.

## Introduction

With a prevalence of up to 84%, low back pain (LBP) is the most prevalent musculoskeletal ailment in adults. When LBP persists beyond 12 weeks, it is termed chronic low back pain (CLBP) [1]. LBP is known to affect both older and younger adults, significantly impacting their quality of life and work performance [2]. The traditional classifications of LBP as acute, subacute, or chronic are being reconsidered in light of evolving understanding, recognizing its persistent and multifaceted nature. While some experience true remission without recurrence after a single episode, many endure ongoing symptoms and disabilities between episodes [3]. CLBP is increasingly recognized as a complex pain syndrome, incorporating neuropathic elements alongside other adverse effects, presenting a formidable challenge due to its variability. Pain receptors, known as nociceptors, are plentiful in various tissues throughout the body, including the intervertebral discs, facet joints, muscles, tendons, ligaments, synovium, and joint capsules, as well as within the intricate network of fascia which envelops and supports these structures. Activation of the nociceptive pathway can trigger pain through a cascade of biochemical processes resulting from the deterioration of these tissues [4]. Weakening or inadequate regulation of movement in the deep trunk muscles may exacerbate CLBP. Individuals with CLBP often exhibit noticeable changes, such as delayed transverse abdominis stimulation, abnormal multifidus recruiting pattern, reduced precision in repositioning, and endurance deficits. Abnormalities in the proprioception of the peripheral proprioceptive system of paraspinal muscles can lead to postural and balance issues [5]. Addressing LBP promptly is crucial as it can lead to further biomechanical alterations over time. Employing optimal management techniques, alongside a range of non-pharmacological interventions, is imperative [6]. Exercise stands out as a widely embraced rehabilitative approach for individuals with CLBP. The primary goals of physical exercise in CLBP treatment include enhancing muscle strength, flexibility, and endurance, promoting tissue healing, and improving the ability to perform daily activities, including work [7]. The components of exercise programs for managing chronic LBP can vary significantly, depending on factors such as exercise duration, intensity, type, and frequency [8]. Proprioceptive neuromuscular facilitation (PNF) emerges as a potential approach for addressing CLBP, involving a variety of manual maneuvers [4]. PNF techniques harness the body's responses and proprioceptive mechanisms to either inhibit or promote muscle contractions. PNF exercises are designed to enhance overall functional capacity, including muscle strength, endurance, joint mobility, stability, neuromuscular control, balance, and coordination [9]. By employing movement sequences, PNF primarily enhances the efficiency of muscles affected by CLBP. PNF treatment utilizes spiral and diagonal patterns of movement, which involve rotational and oblique movements across multiple planes of motion. These patterns have been shown to enhance human performance and alleviate the manifestations of CLBP more effectively compared to traditional single-direction exercise regimens [10]. Other techniques such as rhythmical stabilization and a combination of isotonic methods aim to facilitate trunk motion in seated positions [11]. Strengthening exercises with a Consecutive Loop TheraBand (CLX) (The Hygenic Corporation, Akron, USA) contribute to improved balance and support for the lower limbs [12]. Additionally, elastic band strength exercises have been shown to enhance balance, walking ability, flexibility, strength, and mobility, and alleviate joint pain [13].

## Case presentation

A 24-year-old female, working in a corporate environment, presented with CLBP which was hindering her for five months. The pain showed fluctuations throughout the day, being less severe in the mornings but worsening during prolonged sitting, which was a significant part of her job duties involving both sitting and heavy lifting. The onset of pain was sudden and gradually worsened over time, limiting her ability to sit for long periods and perform routine activities. However, she did not report any radiating leg pain or neurological symptoms. Despite initial treatment with analgesics prescribed by an orthopedician, which targeted paraspinal muscle spasms and thoracolumbar fascia tightness identified during assessment, the pain persisted, leading to a referral for physiotherapy. Upon examination, the patient demonstrated tenderness over the lumbar spine's paraspinal region, along with muscle spasms and fascia tightness contributing to persistent, dull pain rated at 8/10 on the Visual Analog Scale. Specifically, attention was given to palpating for areas of tenderness along the thoracolumbar fascia and surrounding musculature. Additionally, the quality and texture of the fascia were assessed for any signs of thickening, adhesions, or restrictions, which may contribute to the aggravation of the symptoms. Table 1 shows manual muscle testing of lumbar muscles. Sensations and reflexes in both lower limbs were normal. Functional assessments, including the Modified Oswestry Disability Index and Modified Schober’s Test, indicated a significant level of disability attributed to CLBP and limited lumbar flexion, respectively. The negative results of the slump test suggested no neural tension, while a balanced assessment using the Y Balance Test revealed compromised balance. Furthermore, the patient's weight was 60 kg and height was 165 cm. Previous pharmacological intake included an analgesics course prescribed by the orthopedician. However, these treatments did not provide significant relief. Additionally, the patient underwent a radiographic examination of the lumbar spine and laboratory blood tests, which ruled out other organic causes or structural alterations of the vertebrae that contributed to her symptoms.

**Table 1:** Manual muscle testing

Muscles	Pre-rehabilitation	Post-rehabilitation
Lumbar Flexors	3/5	4+/5
Lumbar Side Flexors	3/5	4+/5
Lumbar Extensors	3/5	4+/5
Lumbar Rotators	3/5	4+/5

Physiotherapy management

Table 2 shows phasic intervention for the patient.

**Table 2:** The table shows phasic intervention for the patient IFT: interferential therapy; CLX: consecutive loop exercise; PNF; proprioceptive neuromuscular facilitation; D1: diagonal 1, D2: diagonal 2

Phase	Goal	Intervention
Phase 1 (Weeks 1-2)	Patient education, ergonomic advice	Proper lifting method taught along with dos and don’ts.
	Pain management	IFT for 20 minutes on four pole in sweep pattern.
	CLX adjunct with PNF	D1 and D2 patterns were combined for both upper and lower limbs: First week: In supine position. Second week: Long sitting position.
Phase 2 (Weeks 3-4)	Strengthening	Strengthening and stretching the back muscles involve isometric back exercises, single knee-to-chest stretches, double knee-to-chest stretches, cat and camel exercises, hamstring muscle stretches, piriformis muscle stretches, and quadratus lumborum stretches. Each was repeated 10 times.
	Pain management	IFT for 10 minutes on four poles in a sweep pattern.
	CLX adjunct with PNF	D1 and D2 patterns for both upper and lower limbs: Third week: In kneeling position. Fourth week: Kneeling to standing position.
Phase 3 (Weeks 5-6)	Maintaining achieved strength and increasing strength	D1 and D2 patterns for both upper and lower limbs in standing position.

**Figure 1:**
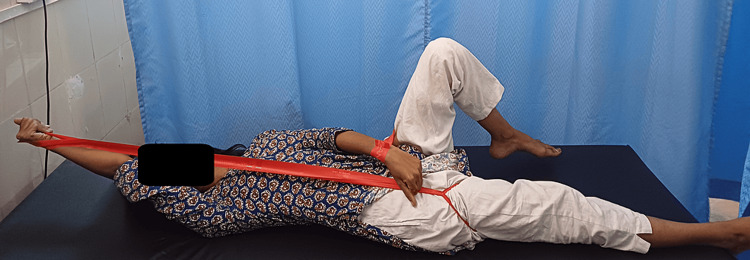
CLX adjunct to PNF in lying position CLX: consecutive loop exercise; PNF: proprioceptive neuromuscular facilitation

**Figure 2:**
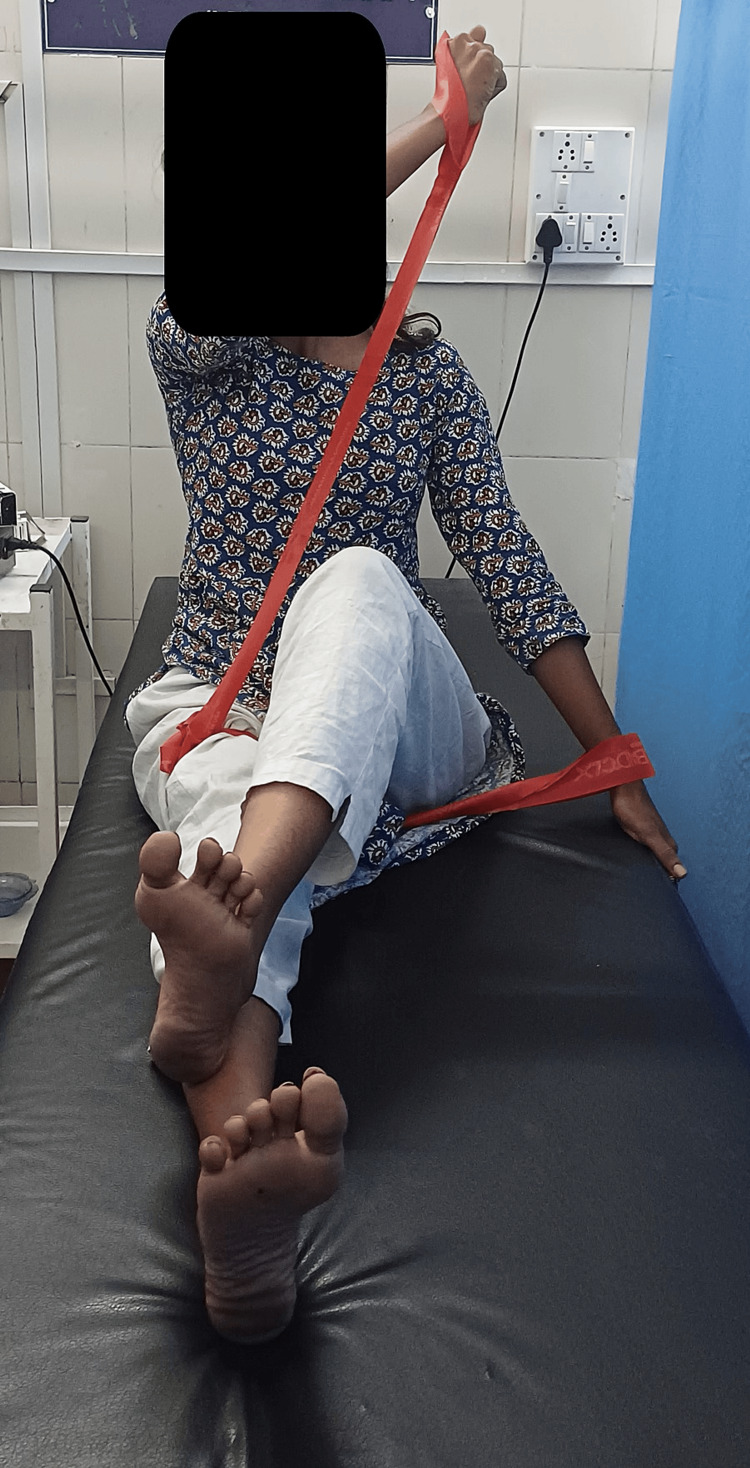
CLX adjunct to PNF in the long sitting position CLX: consecutive loop exercise; PNF: proprioceptive neuromuscular facilitation

Patient education and ergonomic advice were pivotal, emphasizing proper posture, body mechanics, and ergonomic workplace setups. Pain management strategies encompassed pharmacological and non-pharmacological approaches, including heat therapy, cold therapy, and gentle stretching exercises. The CLX adjunct with PNF combined dynamic exercises with CLX bands and PNF techniques to enhance muscle strength, flexibility, and neuromuscular control. Strengthening exercises targeted muscles supporting the lumbar spine, employing progressive resistance training to improve core stability and functional capacity. Maintaining achieved strength and increasing strength involved tailored exercise programs focusing on progressive overload principles and consistency to promote long-term improvements in strength, endurance, and functional capacity. Figures 1-2 show the application of PNF using CLX. And Table 3 shows outcome measures.

**Table 3:** Outcome measures

Scale	Pre-rehabilitation	Post-rehabilitation	Follow-up
Visual Analogue Scale	On activity: 8/10; At rest: 5/10	On activity: 5/10; At rest: 2/10	1/10
Modified Schober’s Test	4.6 cm	5.8 cm	6.1 cm
Modified Oswestry Disability Index	70%	50%	21%
Y Balance Test	75%	82%	90%

## Discussion

CLBP often presents complex challenges like the multifactorial nature of the condition, involving both physical and psychosocial components such as muscular imbalances, biomechanical dysfunction, psychosocial stressors, and lifestyle factors, particularly for younger adults like the 24-year-old woman in this case study. It's evident that there was a notable improvement in pain scores, muscle strength, and balance following treatment and during the two-month follow-up period. The exercise program yielded significant enhancements. Unfortunately, the patient didn't seek physical therapy in a timely manner, resulting in a worsening condition. Hence, there's a need for increased awareness regarding early intervention among the populace. The rehabilitation goals aimed to tackle key areas: reducing pain, improving balance, and ultimately achieving a pain-free state. Moreover, individuals with CLBP may exhibit a reduced ability to adapt to sudden trunk disturbances, indicating a diminished capacity to stabilize in response to abrupt postural changes. Furthermore, they often face difficulties in balance control, potentially leading to challenges in maintaining equilibrium during various activities. Therefore, the rehabilitation approach was comprehensive, not only focusing on pain alleviation but also addressing underlying muscular deficiencies, enhancing adaptability to sudden trunk movements, and improving overall balance to promote a pain-free and functional state for the patient.

Activation of the nociceptive pathway can lead to pain via a series of biochemical processes triggered by tissue degradation [4]. Additionally, abnormalities in the impulse conveyed by mechanoreceptors and deficits in the peripheral proprioceptive system of paraspinal muscles can also contribute to postural and balance issues [5]. The PNF utilizes the body's responses and proprioceptive mechanisms to either inhibit or facilitate muscle contractions. PNF methods can enhance overall functional capacity, encompassing muscle strength, endurance, joint mobility and stability, neuromuscular control, balance, and coordination [9]. Specifically targeting CLBP, PNF exercises enhance muscle efficiency through their movement sequences. Moreover, PNF incorporates spiral and diagonal directions, which have been shown to enhance human performance and alleviate the physical symptoms of long-term LBP more effectively than traditional single-direction exercise regimens [10]. The components of exercise programs for managing chronic LBP can vary, including factors such as the duration and intensity of exercises, as well as the type and frequency of exercise [8]. Hwangbo et al. discovered that PNF techniques target the enhancement of trunk stability and overall body movement by employing diagonal segmental actions in multiple directions. Their study revealed that trunk stability exercises, which strengthen deep abdominal muscles and enhance flexibility and balance capacity, had a more significant impact on daily activities for individuals experiencing persistent LBP compared to combination exercises [14]. Similarly, Cho et al. demonstrated that incorporating PNF patterns into dynamic training effectively improved posture, which serves as the basis for trunk stabilization [15]. Individuals with back pain often exhibit an overreliance on their superficial global muscles, while their control and activation of deep spinal muscles are compromised [16]. PNF operates on the premise that all individuals, including those with impairments, possess inherent capabilities. The primary aim of PNF therapy is to help patients reach their maximum functional capacity. To address muscle wasting, imbalances, joint stiffness, and muscle weakness, PNF employs techniques such as stretching, resisted movements, traction, and approximation. Originally developed for rehabilitation purposes, PNF therapy is effective in restoring strength, range of motion, and flexibility to injured or stiff muscles [17]. PNF methods involve diagonal segmental movements executed in various dimensions, with the objective of enhancing trunk stability and overall body mobility [18].

## Conclusions

Physiotherapy plays a crucial role in managing CLBP by reducing pain, improving strength, and enhancing balance. Early initiation of physiotherapy interventions is essential for preserving strength and managing pain effectively. PNF emerges as a valuable tool in addressing CLBP and associated functional limitations, particularly due to its specialized movement patterns. PNF techniques, including diagonal and spiral patterns, demonstrate superior effectiveness in improving performance and alleviating clinical symptoms associated with CLBP compared to traditional single-direction exercises.
